# Sustainability of Civil Structures through the Application of Smart Materials: A Review

**DOI:** 10.3390/ma14174824

**Published:** 2021-08-25

**Authors:** Alireza Tabrizikahou, Mieczysław Kuczma, Piotr Nowotarski, Małgorzata Kwiatek, Ahad Javanmardi

**Affiliations:** 1 Institute of Building Engineering, Poznan University of Technology, Piotrowo 5, 60-965 Poznan, Poland; mieczyslaw.kuczma@put.poznan.pl (M.K.); piotr.nowotarski@put.poznan.pl (P.N.); 2 UNIBEP S.A., 3 Maja 19, 17-100 Bielsk Podlaski, Poland; malgorzatakwiatek7@gmail.com; 3 College of Civil Engineering, University Town, Key Lab of Fujian Province, Fuzhou University, 2 Xueyuan Road, Fuzhou 350108, China; Ahadjavanmardi@gmail.com; 4 Center of Research and Development, PASOFAL Engineering Group, Kuala Lumpur 52200, Malaysia

**Keywords:** sustainability, civil structures, smart materials, shape memory alloys, piezoelectric, earthquake, building life cycle, review

## Abstract

Every year, structural flaws or breakdowns cause thousands of people to be harmed and cost billions of dollars owing to the limitations of design methods and materials to withstand extreme earthquakes. Since earthquakes have a significant effect on sustainability factors, there is a contradiction between these constraints and the growing need for more sustainable structures. There has been a significant attempt to circumvent these constraints by developing various techniques and materials. One of these viable possibilities is the application of smart structures and materials such as shape memory and piezoelectric materials. Many scholars have examined the use of these materials and their structural characteristics up to this point, but the relationship between sustainability considerations and the deployment of smart materials has received little attention. Therefore, through a review of previous experimental, numerical, and conceptual studies, this paper attempts to draw a more significant relationship between smart materials and structural sustainability. First, the significant impact of seismic events on structural sustainability and its major aspects are described. It is then followed by an overview of the fundamentals of smart material’s behaviour and properties. Finally, after a comprehensive review of the most recent applications of smart materials in structures, the influence of their deployment on sustainability issues is discussed. The findings of this study are intended to assist researchers in properly addressing sustainability considerations in any research and implementation of smart materials by establishing a more explicit relationship between these two concepts.

## 1. Introduction

For decades, studies have widely emphasised on waste generation, environmental degradation, global warming, and economic growth as major issues confronting humanity [1,2]. One of the pillar industries with considerable effect on sustainable advancement and the aforementioned challenges is the construction industry producing up to 25% solid waste each year and a significant share of global economy [3,4]. For example, Europe’s building sector contributing to ~10% of European Gross Domestic Product (GDP) and has significant environmental and social repercussions [5]. Additionally, annually, the construction industry is burdened with exorbitant maintenance and repair expenses for deteriorated and faulty structures, as well as the expenditure of reconstructing them once they collapse due to an earthquake [6]. In Europe, maintenance expenses have reached a high level and are skyrocketing, with monitoring and maintenance operations accounting for the majority of these expenditures [7].

Every year, earthquakes, as one of the severe circumstances exposing buildings, harm thousands of people and cause billions of dollars in property damage throughout the world [6]. In earthquake-prone locations with a large concentration of people and urbanisation, assessing the seismic risk of building stock is a problem for governments and academics [8,9]. The natural hazards approach is essential for establishing disaster response systems and guaranteeing disaster risk and response management in accordance with sustainable redevelopment [10].

**Figure 1:**
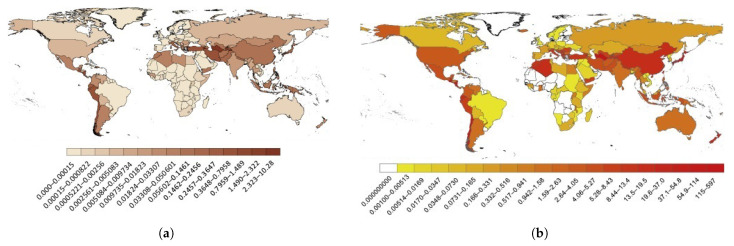
(**a**) Number of yearly earthquake fatalities cumulated in the period 1900 to 2012, split up by population; (**b**) economic loss (GDP) due to earthquakes accrued between 1900 and 2012 at the time of occurrence [11].

The relative risk in terms of the last 113 years of deadly earthquakes may be viewed as a percentage in Figure 1a by normalising inhabitants against the number of deaths in an earthquake. When the GDP of a country at the period of a specific quake is compared to the losses, an accumulated GDP perspective of 113 years of events is obtained (see Figure 1b). As these losses include the cost of maintenance and restoration, a comparative analysis with GDP is feasible.

**Figure 2:**
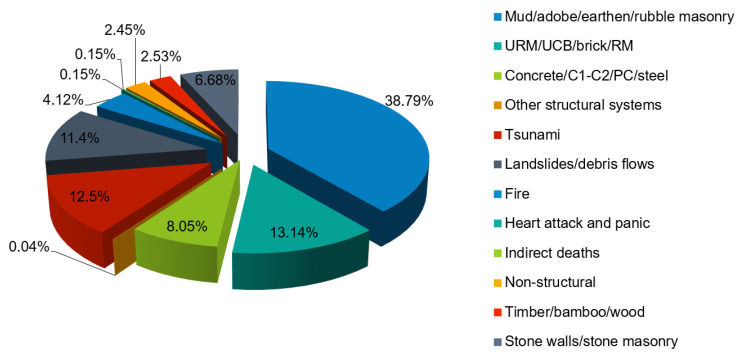
Seismic occurrences and deaths as a result of secondary effects for deadly earthquakes across the world [12].

A thorough examination of the deaths caused by earthquakes has been carried out [12], whether it be a primary structure failure or a subsequent impact such as tsunami, avalanche or otherwise lethal earthquakes in the years 1900–2012. More than 57% of deaths were identified either by collapsing structural components, collapse in the roof, or falling debris in masonry structures (see Figure 2). Slightly less than one-tenth (8.5%) of those died in concrete constructions and 2.53% in timber framing. In all, 71% of deaths occurred as a result of direct earthquakes and 29% as a result of various side effects.

The following points can be interpreted from Figure 1 and Figure 2:

- in countries with low inhabitants and stronger seismic events, the economic losses caused by earthquakes are greater (for example, Turkmenistan, Armenia, and Haiti);
- countries with a massive and dense population (for example, China), not only have the lowest relative danger but also the highest overall risk; and
- a proper structural performance in many types of structural systems would effectively decrease the human and economic losses caused by earthquakes [13].

As a result, one of the primary issues for structural sustainability is lowering costs while improving structural performance with minimal social and environmental repercussions.

However, using traditional design methods, systems and materials suffer from constraints to establishing a sustainable building such as adaptive construction, convenient and simple repairs, structural health monitoring, etc. Engineers seldom address the different aspects of sustainable development in the conventional framework to structure design, which has been affirmed as one of the most significant aspects of the modern integrated building design method [14]. Aiming toward sustainable procedures throughout a building’s life cycle is regarded as a remedy to these issues and transforming societies into sustainable frameworks. The use of ecologically and economically sustainable alternatives in the design of structures is one aspect of introducing this transformation.

Considerations on engineering structures have a significant influence on waste output, water conservation, power consumption, and carbon footprint [15]. Construction methods, suitable building technology selections, practicability, longevity, and other design qualities listed by [16] are the elements of optimal design to achieve sustainability. Adopting ecofriendly materials in the design of a building or structure does not ensure its profitability because addressing environmental issues alone will not result in sufficient sustainability. They must correspond with economic and social considerations [17]. The incorporation of all sustainable concepts into the project life cycle, as well as the participation of all relevant participants, is required for the deployment of sustainable practises [18].

To address these constraints, a plethora of new technologies and approaches for improving the sustainability of civil constructions have been evolved. In general, the objective of these technologies is to develop a flexible structural system with high structural integrity that does not require additional mass, energy, or expenses [19].

**Figure 3:**
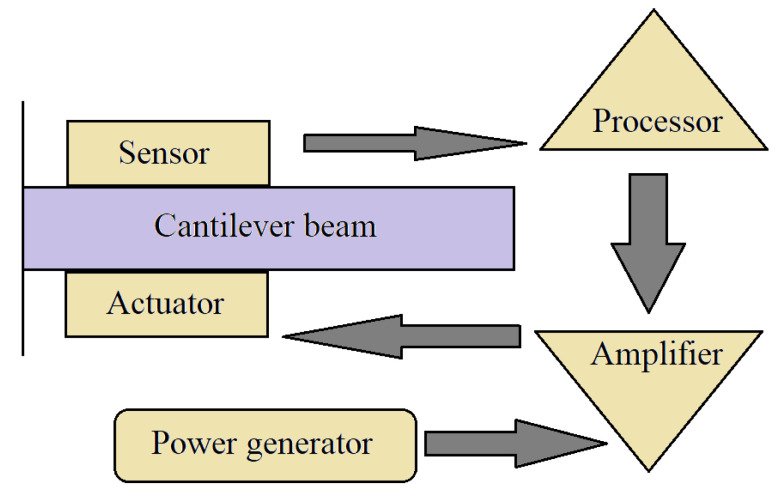
Schematic of a smart structure system [21].

One of these technological advancements makes use of smart structures and materials. The word “smart” refers to these materials’ capacity to automatically sense or detect changes in their environments and respond to those variations through an actuation mechanism (see Figure 3) [20]. Exterior altering surroundings include stresses, energies and geometries, whereas interior changing environments include fractures, breakdowns, and defects.

Smart materials can not only withstand external loads, but can also be integrated with vibration control systems to maintain their superstructure’s integrity and stability throughout its life cycle by altering their shape or mechanical characteristics. Smart materials may be used in buildings to enhance sustainability by directly or indirectly lowering energy consumption, prices, the need for human resources, supervision, etc., while improving structural behaviour, environmental sustainability, etc.

Up to now, numerous scholars have published valuable reviews of the application of smart materials in civil engineering as referenced [22,23,24,25,26,27,28,29,30,31,32,33,34]. However, still, few questions need to be answered as listed below.

- What are the direct and/or indirect effects of the implementation of smart materials on the sustainability of structures?
- What are the scientific and practical gaps of smart materials implementation?
- Is the integrity of interventions for existing structures considered in the employment of smart materials?

Thus, the initial motivation of this study is to answer these questions based on a thorough evaluation of the implications of smart materials for improving structural sustainability. To provide a better grasp of the issue, it is necessary to first present the fundamentals and the principle of structural sustainability regarding the three main factors: environmentally, economically, and socially. Particular attention should be paid to the structural sustainability of buildings and infrastructures in seismically prone areas as they are more vulnerable to external disturbances, which have a major impact on the sustainability elements. As a result, the main focus of this study is to discuss the various types of smart materials and their implementations that increase the structural sustainability of buildings in seismically hazardous places.

Finally, the authors suggested a generic methodology for deploying smart materials based on the evaluation of sustainability aspects. In general, such a generic algorithm would enable civil engineers to obtain a broader view on how to use these sorts of materials; nevertheless, the authors are conscious that this framework must be implemented depending on many aspects in each given structural design system.

## 2. Concept of Structural Sustainability

The two most pervasive descriptions of sustainability are the Brundtland concept, which is identified as fulfilling the requirements that guided its design without jeopardising future generations’ capabilities to meet their own needs [35] and the triple bottom line, which defines sustainability as referring to specific goals: economic, social, and environmental [36]. Sustainable development is described as a condition in which there is an effective balance of social, economic, and environmental factors [37].

The triple bottom line concept of civil structure sustainability considers the three aforementioned major factors. Buildings with poor structural design or performance may suffer significant damage or crumble. This structural damage has an impact on these variables directly or indirectly as a result of their repercussions, which include fatalities, homelessness, repairs, and the discharge of toxic chemicals. Natural catastrophes, such as seismic risks, are often seen as an essential component of sustainability [38].

Sustaining a facility in a hazardous region requires appropriate resilience against natural calamities as, annually, natural catastrophes such as floods, storms, and earthquakes cause multi-billion cost damage to structures. The exorbitant cost of repairing, upgrading, and restoring damaged civil infrastructure has a significant impact on economic growth.

Conventional construction regulations are solely designed for risk assessment in the event of a major catastrophic event. This mindset can lead to a lack of resilience and robustness in structures, which is the primary cause of extensive structural damage. Whereas the effects of natural hazards on civil structures have frequently been overlooked, an increasing effort has been made to link hazard risk minimisation with sustainable structural design. This relationship entails avoiding the damage that impedes sustainability as well as post-event rehabilitation and/or replacement in a more sustainable manner [39].

One such strategy that is already feasible and has been put into partial practice in some locations is performance-based engineering for enduring the severity of earthquake, explosion, and storm impacts. This technique should be recognised with sustainability points as such constructions can reduce the number of energy inputs wasted on repairing defective and or collapsed structures.

Sustainability cannot be accomplished without considering the construction sector’s significant economic, environmental, and social consequences, as well as the poor structural performance. Furthermore, the mending procedures have an impact on the environment by requiring more energy and allowing dangerous chemicals to diffuse as well as other important variables such as fatalities and casualties. As a result of these considerations, effective structural performance in the face of natural catastrophes is directly connected to the long-term viability of civil constructions.

Enhancing a building’s lifespan is greatly advantageous to accomplishing sustainable development as it decreases the proportion of energy consumed per year of demolition and reconstruction. As a result, constructing structures for reliability and durability is a critical structural feature that should be valued in evaluations. Structural longevity is frequently a challenge for bridges, historical buildings, and other civil constructions when the structural system is vulnerable to the surroundings with no insulation. Buildings and structural element endurance have been more of a refurbishment concern than structural stability. As lifespan is a required sustainability feature, structural measures must be developed to improve it. Durability, on the other hand, will necessitate improved environmental factors for a longer life span.

The fundamental structural performances are those that any structure must have to preserve human safety, building functions, and comfort from various forces operating on the building and to sustain the building’s performance for property protection. This guideline specifies safety, reparability, and serviceability as basic structural performances, which relate to the protection of human life, property, and functions, as well as comfort, in the following order [40]:

- safety: to avoid the direct threat to people’s safety both inside and outside the building;
- reparability: to guarantee the ease of restoring structural damages caused by external causes; and
- serviceability: to guarantee the building’s functionality and comfort.

An efficient corresponding to these elements necessitates many measures in a structure’s LC, including materials with superior physical and mechanical properties, continuous inspections and maintenance, and many other processes.

**Figure 4:**
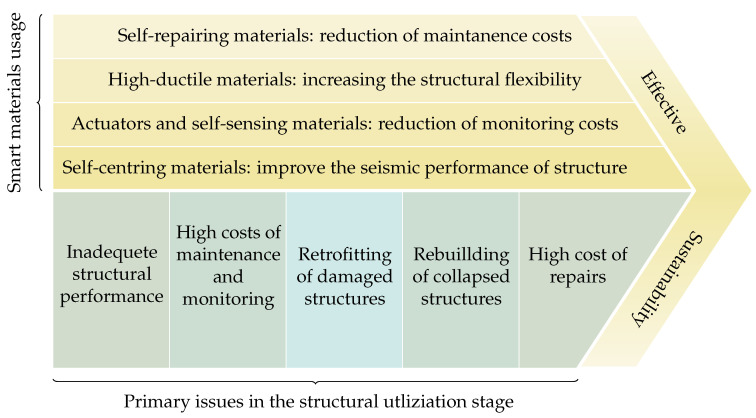
Several advantages of smart materials in the fourth stage (utilising) of a structure leading to an effective sustainable structure.

It is predicted that by implementing such a framework in conjunction with the application of smart materials, the outputs will meet the sustainability considerations. As an example of a successful sustainable application of smart materials to structures, as shown in Figure 4, the advantages of using smart materials are distinctive for each of these sub-stages. As a result, adopting these classes of materials might be regarded as effectively addressing the concerns connected to traditional materials, design processes, and other previously stated problems.

Therefore, it is critical to first become aware of a variety of smart materials, as well as their distinct properties and behaviour. As it is important to examine the features of the material before any deployment in each application, for example, if the criteria demand high strength properties, the selected material should fulfil this requirement. Furthermore, by tracing earlier research and incorporating their findings into current investigations, engineers will be able to acquire more precise results in the application of smart materials.

## 3. Basics of Smart Materials

With the expanding tendency to develop more sustainable constructions with enhanced structural performance, civil engineers have been increasingly interested in smart materials, as they can provide features such as immediacy, self-actuation, flexibility, self-monitoring, and self-healing. Smart materials adapt to the environmental stimulus by adjusting their characteristics, features, and functionalities in a context-dependent, reversible, and repetitive manner via electrical, chemical, and mechanical mechanisms, allowing them to respond to a broad range of configurations and applications. Metals, ceramics, polymers, and composites are the material categories of smart materials in the realm of engineering.

These materials may be classified into several categories based on their behaviour and unique features; however, in this study, shape memory materials and piezoelectrics are solely analysed as they have been the most investigated with high practicality in civil engineering applications.

### 3.1. Shape Memory Materials

Shape Memory Materials (SMMs) are materials that have advantageous features owing to their unique behaviour and properties such as Shape Memory Effect (SME) and pseudoelasticity. Shape Memory Alloys (SMAs) are one of the most attractive groups of SMMs, and they are metallic alloys that can regain their original shape after being subjected to large deformations owing to phase transitions. Shape Memory Polymers (SMPs) are another type of SMMs that may be activated by heat stimulation, magnetic fields, and even light [41].

#### 3.1.1. Shape Memory Alloys

**Figure 5:**
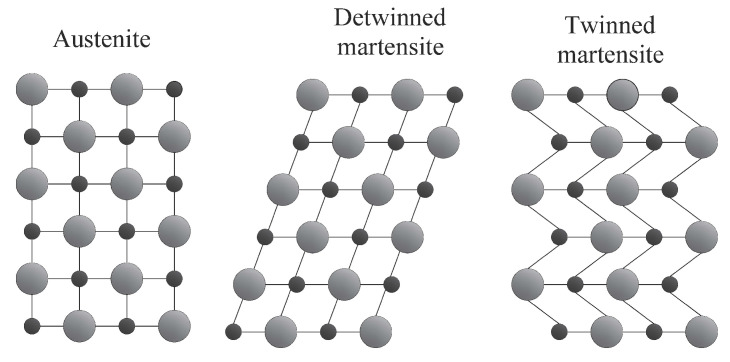
Different crystal structures of SMAs.

SMAs commonly exist in two crystalline forms: the firmer austenite state, which is stable at higher temperatures and lower stresses, and the weaker martensite state, which is stable at lower temperatures and higher stresses. Figure 5 shows that austenite has a body-centred cubic crystalline geometry, while martensite has a trapezoid asymmetric shape with almost 24 distinct configurations. SMAs deform after being subjected to external stresses during the martensite phase due to a detwining mechanism that transforms many martensite variations to a specific form with the maximum elongation. The parallelogram structure of the martensite phase is brittle and sensitive to external pressures. The austenite phase, on the other hand, has just one possible crystalline structure and is highly deformable.

SME and pseudoelasticity are two remarkable properties of SMAs in which SME refers to a material’s capacity to revert to its original shape when heated in its low-temperature phase. Pseudoelasticity is a phenomenon in which SMAs can undergo substantial inelastic deformations before reverting to their normal shape after being unloaded.

**Figure 6:**
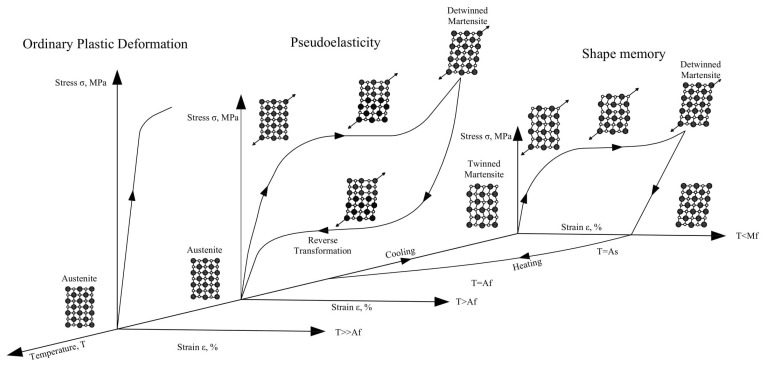
SMA stress–strain graphs depicting schematic crystal formations at different temperatures [26] (figure is taken from [47]).

Figure 6 depicts the unique features of SMAs resulting from reversible martensitic phase transitions. The dissipative character of the deformation process of reversible phase transitions, which constitutes a non-convex issue with hysteresis loops, makes modelling and numerical simulations of boundary value problems for SMA structural components a significant challenge [42,43,44,45,46].

In the absence of applied stresses, the transition from martensite to austenite can occur simply by heating an SMA. The temperature at which the material transitions from twinned martensite to austenite is referred to as the austenite starting temperature ($A_{s}$). The austenite completion temperature is the temperature at which this transformation is complete and the material completely changes to austenite ($A_{f}$). In other words, once the temperature of the SMA climbs over a specific point, its original shape transforms into the austenite structure.

Even with enormous applied forces, this transition phase can occur, resulting in high actuation energy densities. During the cooling process, austenite transforms into twinned martensite at the martensite start temperature ($M_{s}$), and the transition is complete at the martensite completion temperature ($M_{f}$). The transformation temperatures can be affected by changes in the relative amounts of the component metals, the manufacturing process, and the applied stress to an SMA. $M_{d}$ refers to the highest temperature at which martensite can no longer be stress-induced and beyond which the SMA is permanently deformed like any other normal metallic material ($M_{d}\gg A_{f}$).

Up to now, many materials have shown SME and pseudoelasticity behaviour, however not all of them are suitable for civil engineering applications. Because of its high tensile strength, good fatigue and re-centring capability, and a variety of other characteristics, Ni-Ti, copper-based, and iron-based SMAs have shown significant potential for application in civil constructions. As a result, the characteristics and properties of these three types of SMAs will be reviewed and contrasted in the following sections.

##### Ni-Ti Shape Memory Alloys

Ni-Ti has so far exhibited remarkable SME behaviour among the various SMAs, with high magnitudes of shape recovery, recovery stress, and pseudoelastic strain. As a result, structural reinforcement, dynamic response control systems, and structural isolations are among the many applications for Ni-Ti. For decades, binary Ni-Ti alloys and ternary Ni-Ti–X alloys (X denotes potential additional alloying components) have been the most appealing types of SMAs. In contrast to stainless steel, one of the most commonly used materials in civil construction, Ni-Ti as well as Fe-based SMAs have superior properties, as demonstrated in Table 1.

The temperature-induced austenite-to-martensite phase transition, also known as the thermoelastic martensitic transformation, is accountable for Ni-Ti’s memory behaviour. This transition, which leads to shape recovery, is caused by the crystal lattice structure’s demand to maintain a minimum energy equilibrium at a specific temperature. The ability of SMAs to recover a predetermined configuration on heating above the transition temperatures and return to an alternative form of cooling is known as the two-way shape memory effect.

##### Fe-Based Shape Memory Alloys

Because of its outstanding characteristics, such as high stiffness, high strength, and low production cost, this type of SMA has a wide range of applications in civil engineering. Fe-based SMAs are divided into two groups: The first group includes Fe–Pt, Fe–Pd, and Fe–Ni–Co alloys, which have a decent martensitic phase transition behaviour similar to Ni-Ti with low thermal hysteresis. However, no pseudoelasticity has been observed at ambient temperature for Fe–Pt or Fe–Pd alloys.

It has been reported that Fe–29Ni–18Co–5Al–8Ta–0.01B (mass percentage) SMA demonstrates good mechanical behaviour at room temperature, with a recovery strain of more than 13% and tensile strength of 1200 MPa [48]. The Fe–36Mn–8Al–8.6Ni alloy is another exceptional Fe-based SMA with extraordinary superelastic properties at ambient temperature, a recovery strain of more than 5%, and a fracture tensile strain of more than 8% [49]. These Fe-based SMAs have the potential to be very beneficial in circumstances requiring pseudoelasticity and damping capacity. However, because of a lack of mass production advancements and high production costs, these two Fe-SMAs are still not widely used in civil engineering applications [33].

The second group, composed of Fe–Ni–C and Fe–Mn–Si, displays higher thermal hysteresis in transition than the first while still exhibiting SME. Because of their low cost, excellent workability, strong weldability, and other remarkable properties, Fe–Mn–Si SMAs are presently the most appealing iron-based SMAs.

The preponderance of the compositions in mass in most iron-based SMAs is Fe, which is an inexpensive and easily attainable material. As a result, they are a low-cost SMA category with SME and high ductility [50]. These properties include a high pre-stressing strength varying from 13 to 80 MPa at phase transition temperatures ranging from 130 ${}^{\circ }$C to 580 ${}^{\circ }$C. As a consequence, they may be embedded with concrete buildings without causing harm to the surrounding concrete owing to high temperatures upon actuation. Fe-SMAs have different thermomechanical properties than Ni-Ti due to variations in crystal structures and a higher modulus of elasticity [51]. Furthermore, in Fe-SMAs, the rate of recovery strain is related to the temperature at which the martensitic transition occurs.

Dong et al. [52] suggested a low-cost iron-based SMA with high tensile strength, excellent shape recovery stress, and high elastic stiffness as the Fe–17Mn–5Si–10Cr–4Ni–1(V or C). This alloy composition is suitable for structural concrete applications due to its ability to achieve recovery stresses of 580 MPa after heating to 130 ${}^{\circ }$C. As a result, it prevents microcracking and deterioration of the mechanical properties of the surrounding concrete [53]. Furthermore, as compared to Ni-Ti SMA, manufacture is less expensive and simpler, and it can be produced on an industrial scale under atmospheric conditions without the requirement for costly, high-vacuum processing equipment [54].

##### Cu-Based Shape Memory Alloys

Because of their good shape recovery, simplicity of production, and remarkable heat and electricity conductivity, Cu-based SMAs, especially binary Cu alloys with Zn, Al, and Sn with or without ternary additional components, have shown their usefulness. Aside from SME and pseudoelasticity, these alloys also have a crucial feature known as Temperature Memory Effect (TME), which becomes apparent after an incomplete reverse martensitic transition [55].

Due to limitations in thermal stability, brittleness, and mechanical strength, which are directly connected to the microstructure of Cu-based SMAs, they have restricted application in particular conditions. Coarse grain sizes, high elastic anisotropies, and secondary phases or pollutants congregating near grain boundaries, to name a few, for example. Cu-based SMAs would be a better alternative than Ni-Ti alloys in fields other than biomedical because of their lower manufacturing costs, but their commercial availability in civil engineering is currently limited [56]. The most researched copper-based SMAs include Cu-Zn alloys, Cu–Al alloys, and Cu–Sn alloys, despite their poor cold workability and martensite stabilisation. As a result, ternary and quaternary additional components in various amounts have been investigated to improve the mechanical and physical properties of this class of SMAs while lowering commercialisation obstacles. Table 2 compares the properties of the two most commonly used Cu-based SMA to Ni-Ti alloys.

#### 3.1.2. Shape Memory Polymers

When exposed to particular stimuli, such as heat, radiation, electric field, magnetosphere, acidity, certain ions, or enzyme, they can revert to their original morphologies. Thermally sensitive SMP is one of these stimuli that has received a lot of attention and has been used in a lot of different applications. Because of its lightweight, cheap cost, good processability, excellent shape ductility, excellent shape transferability, and customised transition temperature, SMPs outperform SMAs. The following are the major aims for making and developing SMPs composites [57]:

- composites with enhanced mechanical properties;
- as heat conductivity rises, the form recovery induction time decreases;
- SME creation of new polymers and polymer combinations;
- modifying and improving existing SMP mechanical properties; and
- creating SMPs that are triggered by electricity, magnetic fields, light, and moisture.

In general, SMPs outperform SMAs in numerous ways, allowing them to be used in a variety of applications. Table 3 compares these features.

### 3.2. Piezoelectrics

**Figure 7:**
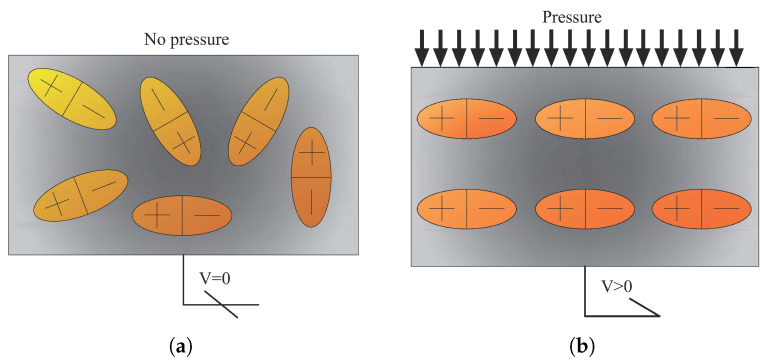
Piezoelectrics materials change their physical shape in response to an electric charge and vice versa; (**a**) before applying the loads (output voltage = 0); (**b**) after applying the loads (output voltage > 0).

The term “piezoelectric material” refers to compounds that display the distinct behaviour of piezoelectricity. When a piezoelectric material is exposed to stress or strain (direct impact), and vice versa, or mechanical deformation, an electric current is produced (Figure 7). As a result, sensors such as accelerometers, sonar, and ultrasonic transducers may take advantage of the direct piezoelectric effect. This behaviour is caused by a primary cell misalignment, a link between mechanical deformation and electron energy divergence.

Piezoelectric materials are available in a multitude of types, each with its unique combination of properties, as below.

- Single-crystal materials: These materials have outstanding piezoelectric characteristics and are commonly utilised as sensors and actuators.
- Lead-based Piezoceramics: Polycrystalline materials with a pyramidal crystal structure that have a high piezoelectric effect and a low dielectric loss. They are, however, very poisonous attributed to the prevalence of lead.
- Lead-free Piezoceramics: These are non-toxic piezoceramics that have a reduced transduction efficiency.
- Piezopolymers are electro-active polymers having properties such as lightweight, non-toxicity, flexibility, biocompatibility, biodegradability and low energy consumption.

Piezoelectric materials provide more sustainable construction elements by dissipating energy from structural deformations and disturbances, resulting in decreased energy consumption and carbon dioxide emissions. When compared to other materials, piezoelectric materials are more adaptable to function in building materials and systems.

## 4. Application of Smart Materials for Sustainable Structures

### 4.1. Application of Shape Memory Alloys

The first reported application of an SMA was in 1969 by the Raychem Corporation, by manufacturing a Ni-Ti-based hydraulic tubing for the US Navy’s fighter aircraft [59]. There are numerous civil engineering applications of SMAs in buildings, bridges and other infrastructures.

Therefore, in this study, the implementations which have a significant impact on improving the sustainability factors have been reviewed thoroughly. These applications are divided into three categories based on the structural systems: reinforced Concrete (RC), steel-based, and masonry structures. SMAs seismic applications comprise passive, semi-active, and active components aiming to reduce the impact of environmental disturbances or seismic loads. Moreover, SMA retrofitting applications applies mainly to the existing buildings with deficiencies in seismic resistance systems (post- or pre-earthquake damaged structures).

The authors are conscious that there have been countless examples and examinations of SMA application in buildings; nevertheless, following a comprehensive assessment of various published studies, the most relevant and newest ones based on sustainability factors have been determined to be examined in this section.

#### 4.1.1. Reinforced Concrete Structures

**Figure 8:**
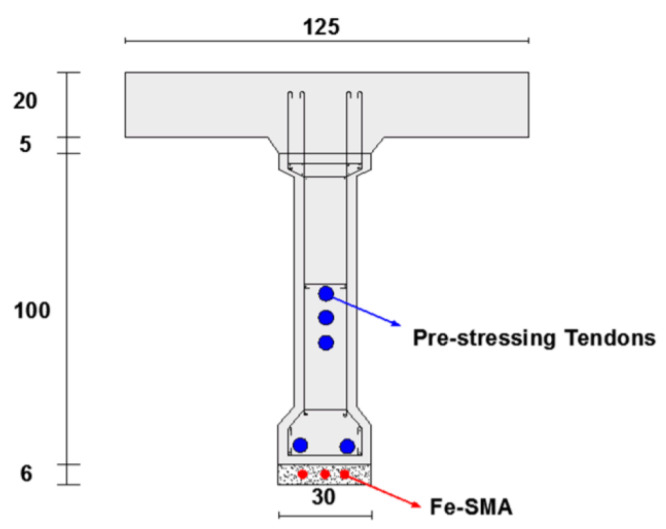
Cross section of the case studied girder (units are in cm) [60].

Dolatabadi et al. [60] demonstrated a method for modelling and analysing the behaviour of RC components strengthened and pre-stressed with Fe-SMA rebars implanted in a shotcrete layer (Figure 8). It was found that employing this technique with a suitable quantity and diameter of Fe-SMA bars increased the load-bearing capability of the bridge girders substantially. It is noted that triggering the recovery stress enhanced the ultimate load by only about 5% when compared to the non-prestressed girder, but it considerably increased the cracking and yielding stresses.

**Figure 9:**
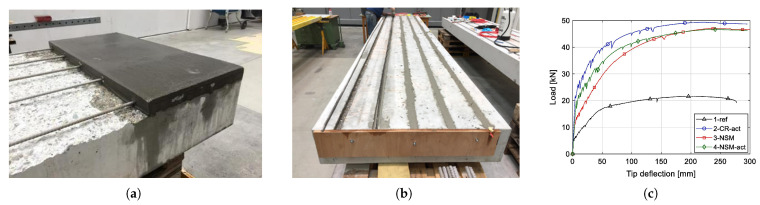
(**a**) First strengthening method; (**b**) second strengthening method; (**c**) load–tip deflection diagram of slabs during quasi-static loading [61].

Schranz et al. [61] use Fe-SMA bars to examine the efficacy of two alternative prestressed strengthening techniques for concrete members in flexure. The first technique of reinforcing involved removing the concrete cover and installing SMA bars with an extra mortar layer (Figure 9a). The second technique of reinforcement involved carving grooves in the concrete cover, followed by the insertion of SMA bars with mortar (Figure 9b).

All of the examined strengthening techniques were shown to considerably enhance the fracturing, yielding and ultimate loads, as well as the ductility of the RC slabs. Figure 9c graphically shows the influence of prestress on performance under quasi-static loading.

**Figure 10:**
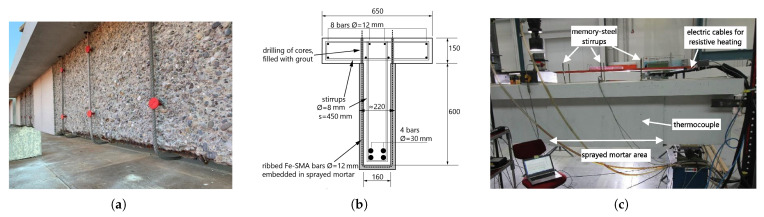
(**a**) Employment of the SMA stirrups on the RC beam; (**b**) cross section of the reinforced RC beam; (**c**) the test installation [62].

Czaderski et al. [62] investigated the use of novel U-shaped Fe-SMA ribbed bars in conjunction with sprayed mortar for shear reinforcement of RC structures (Figure 10). When the SMA stirrups were prestressed, the stress level in the internal steel stirrups was reduced. It is especially beneficial for applications in infrastructure constructions that are subjected to cyclic loads and the associated fatigue concerns. Furthermore, prestressing the SMA stirrups increased the load at which shear fractures appeared.

**Figure 11:**
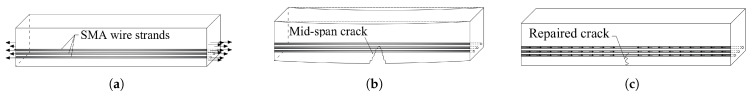
Schematic of intelligent RC specimen: (**a**) RC beam downside is under tension; (**b**) Micro-cracks appear in mid-span; (**c**) Activated SMA wire strands upon heating reduce the micro-cracks [63].

The Intelligent Reinforced Concrete (IRC) approach presented by Song et al. [63] is focused on the actuation characteristic of post-tensioned SMA strands. The IRCs have numerous features, including self-rehabilitation, self-damping, and self-structural health monitoring (SHM). The materials that may exhibit these characteristics are primarily piezoceramics and SMAs. They used post-tensioned martensite SMA rods to retrofit the buildings. After fractures form and expand in an RC element, annealing the SMA rods allows these cracks to nearly entirely merge and mend (see Figure 11). The initiation and depth of fractures in the structure are determined by implanted activated piezoelectrically.

**Figure 12:**
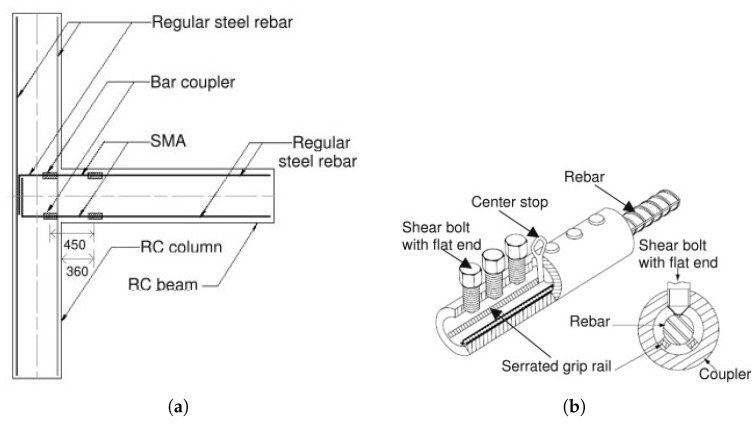
(**a**) Reinforcement details of specimen; (**b**) Splice details of specimen [65].

Youssef et al. [64] examined two large-scale beam–column junctions with inverted cyclic pressure. The first junction was strengthened with normal steel rebars, whereas the second was strengthened with SMA at the plastic hinge zone of the beam and conventional steel in the remaining section of the junction. In the second specimen, the Ni-Ti alloy rebar was employed as reinforcement. Figure 12a depicts the precise design of the couplings. Figure 12b displays the splicing connectors utilised in the reinforcement shackling of the second specimen.

The computational findings anticipated by the FE model correspond substantially with the actual data, differing by just 5.6% for the base shear and 6.1% for the peak deformation. The total energy absorption estimated from the anticipated load–displacement graph was 48.2 kN m, while the total energy dissipation acquired from the practical outcome was 44.0 kN m, which is just 9.4% less than the numerical results.

**Figure 13:**
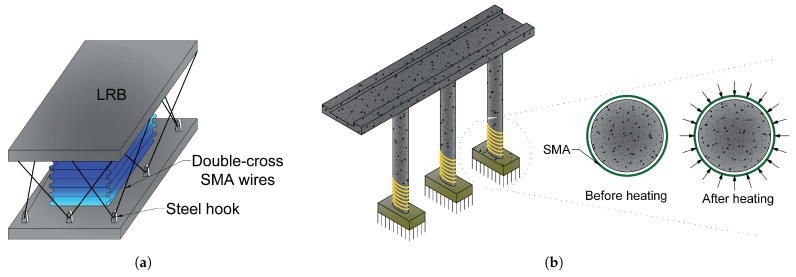
(**a**) SMA wire-based lead rubber bearing [66]; (**b**) The system of utilising external confining loading on RC bridge columns [68].

Dezfuli and Alam [66] designed a novel SMA-based Lead rubber bearing (LRB) by stringing Ferrous SMA wires in an asymmetrical double cross arrangement around the LRB.(Figure 13a).

When compared to traditional LRBs, the suggested SMA-LRB had a much reduced shear strain requirement (up to 46% reduction) and a higher energy dissipation capacity (up to 31% increase). Furthermore, the pseudoelasticity behaviour of Fe-based SMA (above 13% strain) improves the re-centring capacity of LRB by lowering residual deformation by up to 33%.

Billah and Alam [67] performed a stochastic performance-based risk evaluation of five SMA-RC bridge pillars with different SMAs when exposed to three distinct seismic conditions (crustal, in-slab, and interface), all of which influence considerably to Vancouver’s seismicity. Figure 13b displays the adopted mechanism in this study.

The findings demonstrate that at the highest estimated earthquake magnitude, all of the SMA-RC pillars had a very negligible likelihood of collapsing. It was discovered that the bridge pillar strengthened with Fe-N-C-A-T-B-outperformed the other SMA-RC pillars.

#### 4.1.2. Steel-Based Structures

**Figure 14:**
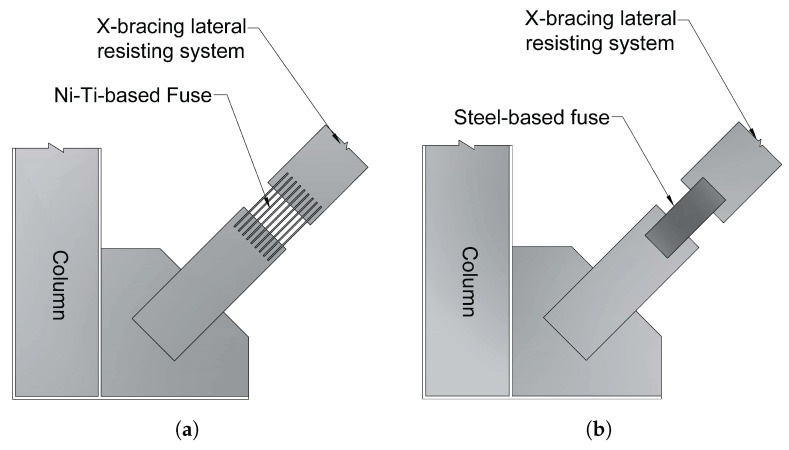
(**a**) Bracing frame with steel-based yielding fuse; (**b**) bracing frame with SMA-based yielding fuse [69].

Varughese and El-Hacha [69] studied the efficiency of a steel-framed structure equipped with a steel resisting fuse and a Ni-Ti SMA fuse (see Figure 14). The results showed that the stiffness of the bracing system does not affect the natural frequency of the frame system but rather that the system’s coupling to the rigid floor modifies the behavioural pattern.

These frames exhibit the equivalent in-plane eigenfrequency according to the results of the experiments (Table 4). It signifies that the fastening at the pillars, rather than the stiffness of the restrained components, dominates the system’s stiffness. As the NiTi-SMA bracing contains approximately 22% of the stiffness of the steel brace, the frequency of the NiTi-SMA brace should be larger than the frequency of the steel brace. The experimental stiffness of both systems are quite close to one another, showing that the frames behave similarly to rigid frames. As a consequence, the stiffness of the braces does not affect the frame’s inherent in-plane behaviour.

**Figure 15:**
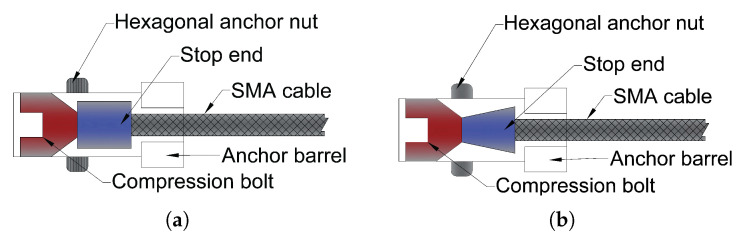
(**a**) Schematic of first SMA-based anchorage system; (**b**) schematic of second SMA-based anchorage system [70].

Shi et al. [70] suggested two distinct SMA cable anchoring systems: a spike-type anchorage system and an end stop-type anchorage system, both of which rely on mechanical anchorage (Figure 15). Both anchoring methods demonstrated extremely excellent pseudoelastic behaviour under all loading circumstances, as well as good performance under high rate and cyclic loading, according to the experimental data. When used in seismic control devices, SMA cables should be anchored with an end stop anchoring mechanism.

The first sample burst with an ultimate strain and stress of 9.9% and 792 MPa, respectively, while the second specimen had an ultimate strain capacity of 13.5% and ultimate strength of 1284 MPa. During the test, the first specimen experiences many sudden strength reductions. Drops form when the strain magnitudes are 2.2%, 3.3%, 3.5%, 4.7%, and 9.5%. In the second specimen, strength reductions occur at strain amplitudes of 12.2%, 12.7%, and 13.3%, respectively, and the SMA cable fails.

**Figure 16:**
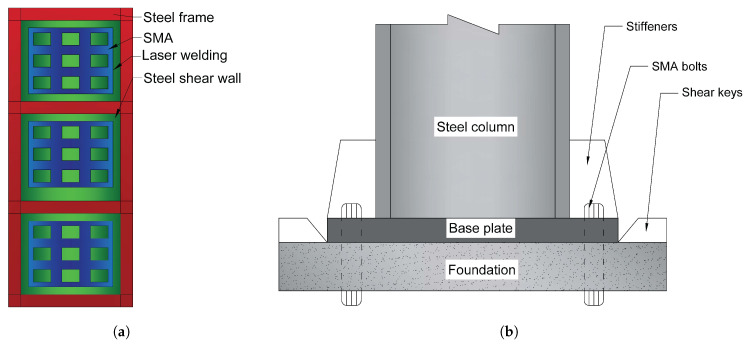
(**a**) The steel structure reinforced with steel shear walls and SMA fibre strands [71]; (**b**) Schematic of steel column and foundation connection with SMA bolts [72].

Kamgar et al. [71] investigate the effects of SMA on Steel Shear Walls (SSW). They introduced SMA fibres diagonally and longitudinally to several sections of the SSW, as shown in Figure 16a. The results show that refurbishing with the SMA reduces the out-of-plane distortion of the SSW under periodic and seismic loadings (Table 5. Consequently, the buckling load increases once the SSW is retrofitted with the SMA. The SME behaviour of SMAs also influences the SSW’s total out-of-plane deformation and nonstructural damage.

Wang et al. [72] investigated the mechanical behaviour of high-performance self-centring steel columns utilising two distinct (the second sample was pre-strained before testing) specimens of SMA-based bolts (Figure 16b). The SMA bolt-equipped column-foundation connection exhibited moderate and stable flag-shaped hysteresis loops with excellent self-centring and appropriate energy dissipation capabilities. Furthermore, the bolts do not need to be changed after large earthquakes and continue to function in the case of aftershocks or subsequent earthquakes.

Both specimens could preserve a maximum drift proportion of 3%. After the 2% targeted drift intervals, both columns showed little residual drift (the maximum residual drift ratio was 0.25% of the first sample). Furthermore, even after being unloaded from 3% drift, the second sample showed little residual drift because pre-strain in the SMA bolts and longitudinal compressive stress in the column may improve the column’s self-centring capability. More importantly, even when the lateral force was removed, the SMA bolts with pre-strain remained securely in place. As a result, the proposed self-centring column should facilitate a seismic resilience structure that demands no (or minimal) maintenance even after massive earthquakes while being utterly capable of aftershocks or following earthquakes. The ductility ratios for the first and second specimens were 3.8 and 7.9, respectively.

Liu et al. [73] investigated the seismic behaviour of a superelastic SMA spring in a multi-story steel frame isolation mechanism. Their findings revealed that the SMA spring has outstanding self-centring and damping capacity, with an equivalent damping ratio of more than 2%. In comparison to typical elastic springs, their SMA spring enables superior control of the superstructure’s maximum and residual deformations.

**Figure 17:**
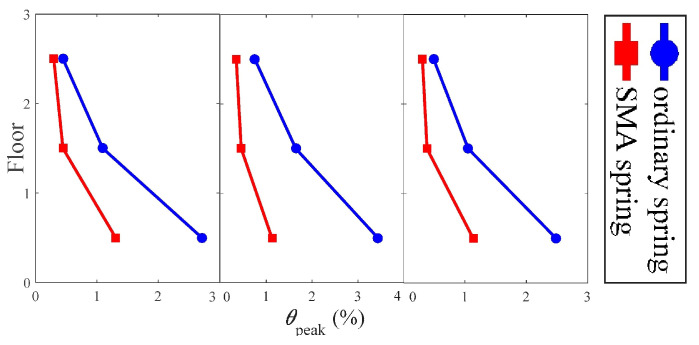
Peak interstory drift ratio of the superstructure under three different earthquake motions [73].

Figure 17 indicates that the peak inter-story drift proportions tend to concentrate at the first storey for all examined seismic activity, however the deformation concentration degree is substantially reduced by using SMA springs. In terms of deformation control throughout the full building height, SMA springs beat conventional springs. The peak inter-story drift ratios of the frames separated with regular springs are approximately 2.7%, 3.4%, and 2.5%, respectively, while the apex inter-story drift ratios of the frames isolated with SMA springs are approximately 1.3%, 1.1%, and 1.1%, respectively, under the three different ground motions. In all cases, the corresponding savings are projected to be more than 50%. It demonstrates SMA springs’ advantage over conventional springs in the isolation system of multi-story frames.

#### 4.1.3. Masonry Structures

Historical constructions must be maintained due to the deterioration of masonry, mud, and earthen building materials; lengthy exposure to climate conditions; and uneven settlements. Furthermore, many historic structures were built to endure gravity stresses first and foremost, or at a considerably lower seismic intensity than similar modern structures [74,75]. The seismic retrofit techniques must then be incorporated into the structure without detracting from its aesthetics. However, the structural components required to absorb the out-of-plane horizontal thrusts created by arches, vaults and wooden roof trusses are frequently absent, inadequate, or deteriorated [76].

Seismic retrofitting approaches for masonry structures may be broadly characterised as follows:

- minimising the seismic pressures that may be applied on the structure;
- increasing the current building’s resistance to earthquake load by structural system changes or upgrading the components’ strength.

Cultural heritage assets, which have a range of characteristics such as historic, social and psychological significance, are critical for attaining urban sustainability [77,78]. As a result, one of the engineers’ primary goals has been to improve the earthquake resilience of these sorts of constructions.

**Figure 18:**
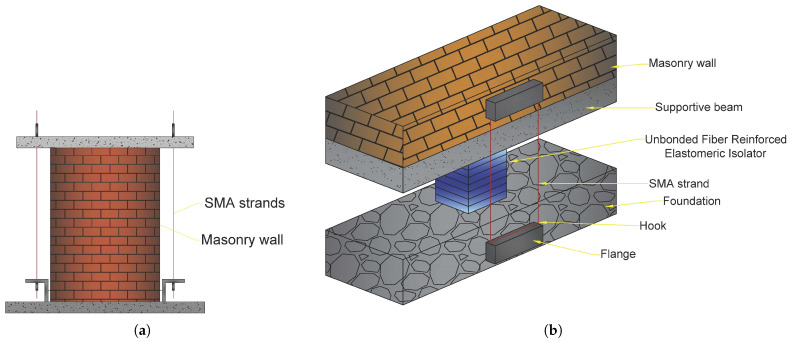
(**a**) The schematic of the proposed system with SMA strands for connection bonds [79]; (**b**) the schematic of the proposed system and SMA strand in the isolation system of masonry constructions [80] (figures are taken from [47]).

Casciati and Hamdaoui [79] examined the implementation of pre-tensioned SMA strands to rehabilitate old masonry buildings. They examined the structural features related to the usage of an SMA-based connection bonds device, as illustrated in Figure 18a.

The results of experimental research were employed to create a numerical model that incorporates the effects of SMA devices. The structure was initially studied in its original state, with no retrofitting attempts and then the effects of several retrofitting techniques were experimentally evaluated and included in the numerical analysis by altering the equivalent Young modulus accordingly.

They evaluated multiple parameters between the use of SMA and steel wires under 20% of the El-Centro earthquake. These parameters encompass frequencies and maximal deformation in systems with and without reinforcing wires (SMA or Steel based). Likewise, the equivalent modulus of elasticity ($E_{eq}$) of the system without any strand was 5875 MPa, whereas it was 19,180, 20,665, and 22,100 MPa for the systems with 2, 4, and 6 SMA wires, respectively.

Habieb et al. [80] studied the use of an embedded base isolation system for seismic protection of a historic brickwork church. The system was built with an unbonded fibre reinforced rubber isolator and SMA cables (see Figure 18b). Due to its high energy absorption capacity, the suggested model with a 2% pre-strain SMA wire model demonstrates the biggest reduction of the lateral deflections of the church and significantly lowers impact (from destruction to mild damage level) in the event of powerful earthquakes.

Note that almost all of the fixed-base model’s reference points have drifts larger than the limit value linked with the breakdown grade. The deflections from collapse to levels of minor/moderate damage are substantially reduced by the base-isolated models. The pre-strained SMA-based model exhibited the largest decrease in deflections among the base-isolated models. The residual deformations in the non-pre-strained SMA-based model, on contrary, are rather large.

**Figure 19:**
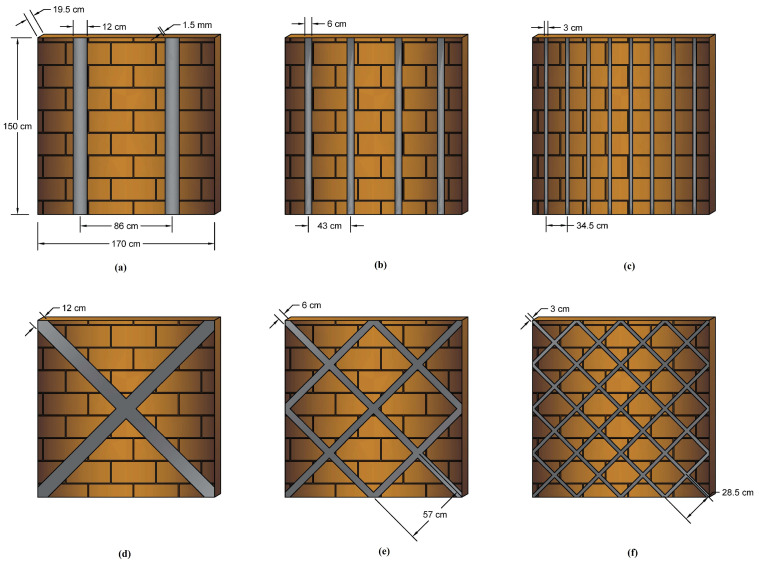
Numerically studied SMA-reinforces walls [81]: (**a**) 2 vertical stripes; (**b**) 4 vertical stripes; (**c**) 8 vertical stripes; (**d**) 2 cross-strips; (**e**) 6 cross-strips; (**f**) 14 cross-strips (figures are taken from [47]).

Rezapour et al. [81] investigated the performance of URM strengthened with iron-based SMA bars. Figure 19 shows SMA-based strips installed in the configuration of crossovers and parallels in brickwork and exposed to post-tension stress with cement as the interface between the wall and the stripes.

The outcomes of this investigation showed that the stiffness rose by 98.1% in the vertical-strip walls and by 127.9% in the crossover model’s location. In addition, the highest resistance in the parallels configuration was 108 kN, although, by the end cycle, it had been decreased by over half to 40 kN. The larger the dispersion of the iron-based strands in massive deformations, the more resistant the brickwork wall. As a result, in model (f), the highest resistance is 104 kN and in the last cycle, it falls by just 13.5% to 90 kN.

**Figure 20:**
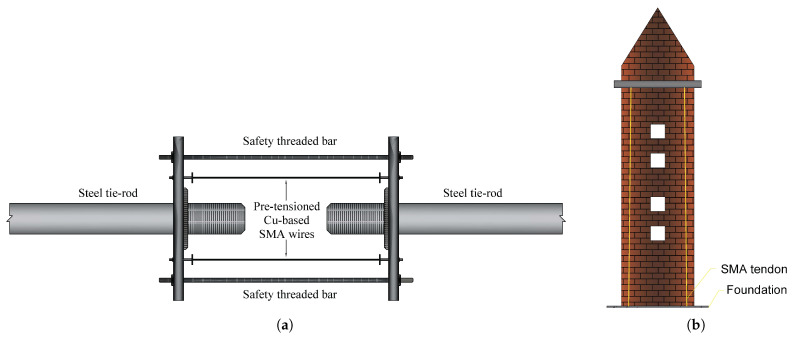
(**a**) Retrofitting scheme of the San Paolo Eremita church [76]; (**b**) installation of four pre-tensioned steel tie bars and SMA devices in the interior corners of the S. Giorgio church bell tower that were anchored to the foundation and the roof [82] (figures are taken from [47]).

Cardone et al. [76] suggested a method based on the superelasticity characteristics of copper-based SMA to enhance the thermodynamic and seismic behaviour of steel connections in ancient structures (San Paolo Eremita church). Figure 20a depicts the proposed model, which connects the two steel tie-rods with pre-tensioned copper-based SMA strands. The results of the experiments demonstrate that the proposed device is successful at minimising force variations induced by changes in air temperature. The results of the tests clearly show that the suggested SMA-based device is highly effective in enhancing the thermal behaviour of steel connections. Indeed, the force variations in the steel tie-rods induced by air temperature variations are 80–90% lower with SMAs than without.

During the tests with the synthetic ground motions at 0.6 g, the hysteretic performance of the connector with SMA device was congruent with the strain boundary of SMA (equal to 9–10%, comparable to the end of the martensitic transformation) and yield force of the steel tie-rod, equivalent to approximately 3 KN. Furthermore, the system with the SMA unit demonstrated outstanding recentring capability. The tie-rod without an SMA device, on the other hand, experienced severe plastic deformations with significant residual deformations of the magnitude of 21 mm after the earthquake, equivalent to 2.4% residual deformation in the steel rod.

Castellano et al. [82] assessed the utilisation of SMA in seismic retrofitting of heritage landmarks. The SMA-based technique was employed in the rehabilitation of the Bell-Tower of San Giorgio at Trignano, with four pre-tensioned SMA devices at the tower’s extremities (see Figure 20b). The feasibility of using SMA devices with various behaviours was demonstrated by the construction of several prototypes that were thoroughly evaluated. Shaking table tests indicated that a new binding technique based on SMA devices can be highly effective in avoiding out-of-plane disintegration of exterior masonry walls, church façades and buildings that are poorly connected at ground level.

It was discovered that the SMA equipment provides considerable acceleration attenuation, for instance, nearly 50% at the peak and more than 60% at the level of the interconnection. Likewise, the maximum force apex in the SMA-reinforced wall is decreased by 45%. When compared to conventional anchors, SMA device ties can increase the permeability of such masonry walls against out-of-plane seismic vibrations by at least 50% (in terms of maximum PGA tolerable without deterioration), leading to a 50% drop in peak acceleration. Moreover, despite ordinary steel connectors, SMA device connections may prevent tympanum global damage. Pseudo-dynamic, in-plane tests on masonry wall sneer with cavities indicated that SMA devices used in conjunction with steel cables to prestress the masonry may absorb about 30% of the seismic energy produced and therefore enhance a structure’s seismic resistance.

### 4.2. Application of Piezoelectrics

**Figure 21:**
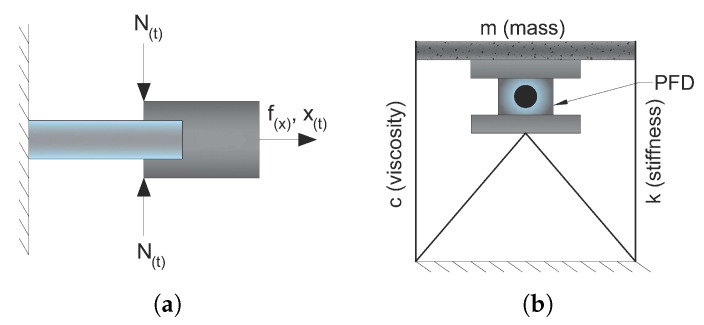
(**a**) Schematics of a semi-active piezoelectric friction damper; (**b**) installation of a damper on a single-story building [83].

Piezoelectric Friction Damper (PFD) is a type of semi-active energy dissipation control device. Chen and Chen [83] proposed a PDF installed between a floor of a building structure and the supporting bracket with the contact force adjusted by piezoelectric stack actuators (see Figure 21).

It is comprised of two components that slide against each other. The sliding surfaces’ force acting, $N_{\left(t\right)}$, is adjustable. The friction damper produces a dissipative force proportionate to the contact force and friction coefficient between the two components. It is feasible to optimise the dynamic behaviour of structures that use friction dampers by changing the contact force depending on damper slippage input. One method of controlling the contact force is to utilise piezoelectric layer actuators to create the necessary force based on control algorithms.

They evaluated the free vibration and dynamic responses of a single-story frame structure integrated with a PFD analytically and observed that the piezoelectric friction damper may significantly lower the structure’s response. According to the research, the PFD technology considerably decreased the maximum responses, and therefore the damper enhanced the damping capacity of a Single Degree of Freedom (SDOF) system.

**Figure 22:**
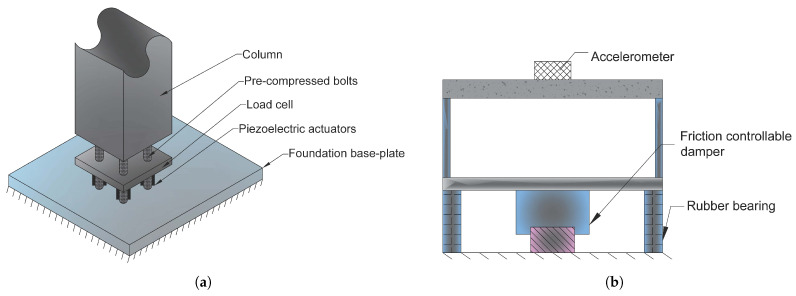
(**a**) Actuator configuration at the bottom of the column [84]; (**b**) structure model of the semi-active base isolation system [87].

Aizawa et al. [84] and Kamada et al. [85,86] represented the use of 32 piezoelectric stack actuators for dynamic response control of a four-story frame, by embedding the actuators into the bottom of a column. They intended to generate a deflection by installing four piezoelectric actuators at the base of a column, as shown in Figure 22a. The piezoelectric actuator is pre-loaded with biassed voltage, enables it to adjust the length from a biassed state length with changing voltage. Four PZTs operate in two pairs and the bending moment is generated by the motion of one pair extending and the other pair shortening simultaneously.

The results showed that the maximum response of the controlled case is about 12.5% of the non-controlled case. Furthermore, the maximum acceleration in the controlled case is about 33.33% of the non-controlled case and the root mean square value is ~25%. As a result of the closed-loop control, 10% of first- and second-mode damping ratios were observed.

As illustrated in Figure 22b, Morita et al. [87] presented a semi-active base isolation system with adjustable friction dampers and piezoelectric actuators for structural response management.

They demonstrated that the response displacement of the structure could be decreased up to 50% of values of the passive control isolation system without limiting the isolation efficiency. It is also reported that the maximum acceleration frequency of the roof might be decreased to 60% of passive insulation values and the maximum relative movement value could be minimised to 50% of passive insulation values.

**Figure 23:**
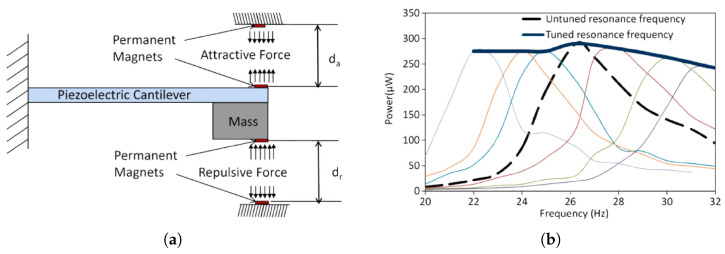
Frequency tunable harvester with geometrical tuning proposed by Challa et al. [88] (figure taken from [89] with permission); (**a**) schematic design of the proposed harvester; (**b**) frequency response for different adjustment.

Challa et al. [88] demonstrated a piezoelectric harvester with a magnetic adjusting mechanism. The suggested device is composed of a vibrating structure with two magnets at its free end and two magnets placed at the enclosing, as seen in Figure 23. The cantilever is supported by a laterally moveable grip that enables the spacing between both the magnets on its end and the anchored magnets to be adjusted.

By modulating the spacing, the magnetic forces may be altered, providing the resonating frequency to be controlled. The resonance frequency of the unamplified device is 26.2 Hz, while no electromagnetic forces are imposed, although this is then decreased when the cantilever beam is adjusted toward the favourable magnetic field and boosted when transferred toward the repelling magnetic field. The circumstance in which the magnets come into encounter limits the deflection. The system’s frequency response, shown in Figure 23b, demonstrates cantilever beam frequency shifting down to 22 Hz and up to 32 Hz.

**Figure 24:**
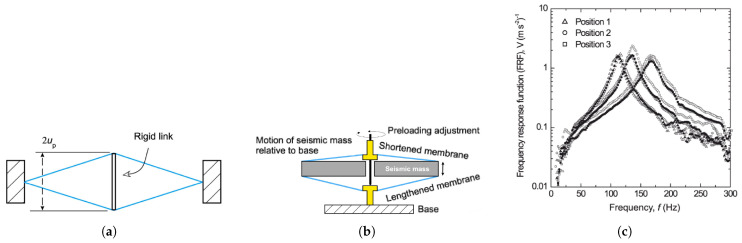
Frequency tunable piezoelectric XMR harvester proposed by Morris et al. [90] (figure taken from [89] with permission); (**a**) basic concept (without seismic mass); (**b**) cross-sectional schematic when driven by base excitation; (**c**) frequency response for different positions.

Morris et al. [90] presented a novel technology based on the elongation of a cantilever instead of bending. Figure 24a depicts the proposed eXtensional Mode Resonator’s operating mechanism (XMR). A disk-shaped seismic bulk with two piezoelectric plates interconnected at their centre by a 2$u_{p}$ length stiff connection forms the concept. These two sheets are fastened to the seismic bulk at their upper and bottom ends with anchoring hooks (see Figure 24b).

Once an exterior stimulation develops, the friction caused by seismic mass disturbances generates transmembrane extensional/contractive displacement. PVDF-based components are mounted in the middle by hinged metal spindles joined by a bolt that runs through the seismic mass’s opening. Tightening the bolt enables to modify the spacing between the centres of the films, which is originally equivalent to $u_{p}$ and thus tune the resonant rate $f_{n}$. The linked system demonstrated a linear connection between $f_{n}$ and the pre-deflection of the extensional components up, which was provided by

(1) $$f_{n}=u_{p}\frac{1}{2\pi }\sqrt{6\frac{Ewh}{ml^{3}}}$$

where *E* represents Young’s modulus, *w* represents the width, *h* represents the depth, *m* represents the seismic mass, and *l* represents the half-length from one side to another. They suggested frequency adjustable devices that rely on this equation, as illustrated in Figure 24c. As a result, a configurable connector that proportionately pre-tensions both piezoelectric plates enables the device frequency programmable. Beginning from an arbitrary starting stance 2, the distance $u_{p}$ is dropped by 0.1 mm for position 1 and raised by 0.2 mm for position 3. The frequency response of the system, shown in Figure 24c, exhibits varied resonance frequencies of about 110, 130, and 160 Hz for positions 1, 2, and 3, respectively.

## 5. Results and Discussion: Sustainable Implementation of Smart Materials

After investigating the methodologies and findings of the preceding research, based on the literature review, it is reasonable to conclude that the use of smart materials enhances structural performance adequately. As a result, the sole concern would be whether the use of these materials would properly fulfil the sustainability standards, therefore, it is critical to consider all of the economic, ecological, and social implications of using smart materials in civil constructions.

Note that smart structures mostly comprise sensors, actuators, and smart materials that exhibit several unique properties that can improve structural performance. Properties such as high strength, self-sensing, self-repairing, self-actuation, and self-centring. These properties make smart materials and systems as the potential for more suitable civil structures. One of the classes of smart materials is magnetostrictive materials with the ability to change magnetic energy into kinetic energy, and vice versa. They have mostly been used as actuators and sensors for buildings. Piezoelectrics as another group of smart materials can become electrically charged when directed to mechanical stresses, and vice versa. Moreover, one of the most attractive groups of smart materials is SMAs which have been used widely in different civil engineering applications. SMAs can undergo large deformations and then return to their original shape by subjecting them to a specific stimulus.

This review study is intended to enable future research to better establishing the correlation between sustainability issues and the application of smart materials. The following are some of the study’s practical implications which might fulfil the three factors of sustainability:

- low-cost SMAs (Fe- and Cu-based), as well as SMPs, should be considered to be used in the structural design of the construction for an affordable implementation of smart materials,
- the environmental consequences of using smart materials should be addressed carefully, as exposing Ni-Ti SMAs to a severe corrosion environment, for example, might result in the release of Nickel, a very poisonous chemical,
- while contemplating the refit of a defective structure, it is critical to consider the social implications; for example, if a bridge requires more retrofitting with smart materials, alternate modes of transportation must be given to avoid disruptions in traffic and people’s lives.

**Figure 25:**
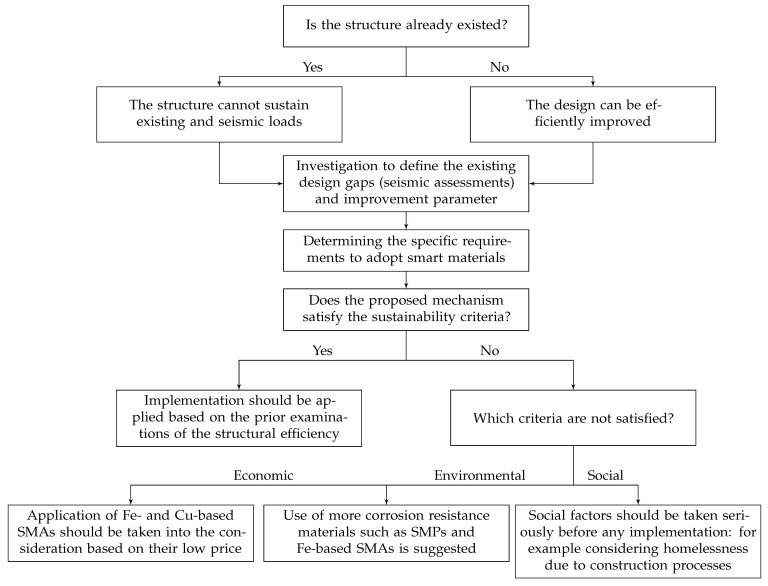
A generic algorithm for using smart materials maintaining sustainability factors.

In addition, a generic algorithm, depicted in Figure 25, is presented by authors to optimise structural sustainability through the use of smart materials.

Because smart materials may be utilised in the execution and retrofitting of structures, the kind of structure must first be defined: existent or intended to be created. Retrofitting structures has a major influence on sustainability aspects as older or post-earthquake-damaged earthquakes often require further repairs. These effects are determined by their historical significance or vital infrastructures, such as buildings or power plants. Nonetheless, by incorporating smart materials into the design of new buildings, future maintenance and repair costs and energy usage may be significantly reduced.

## 6. Conclusions

A structure may be considered sustainable by adequate responding to three main factors of suitability; economic, social, and environmental impacts. In other words, it has to be environmentally responsible and resource-efficient throughout its life cycle. Conventional materials such as steel and concrete and design methods in civil structures have shown weakness by subjecting them to severe seismic forces. It can lead to damaged or collapsed structures which cause outrageous repairing costs, causalities, environmental contamination, etc. This is why it is necessary to improve structures’ performances by more adaptability and flexibility throughout a seismic event. It will lead to more suitable structures that can withstand earthquakes while minimising the deflections, further repairs, and causalities.

The proposed algorithm, which is a concept for the use of smart materials in sustainability improvement, provides promising and broad ideas for academics interested in researching the topic of sustainability, smart materials, and the building life cycle. It is especially essential nowadays that LCA assessments are emerging common procedures in construction as they result in waste and energy reduction during the life of the building. The notion necessitates additional research and the authors want to create and study various case studies, notably for the use of low-cost smart materials, which reduces project costs.

This study provided a brief review of the application of smart systems and materials in civil engineering. In particular, the applications of shape memory alloys such as self-repair, self-sensing, and retrofitting, and piezoelectrics applications such as piezoelectric friction damper, piezoelectric-based semi-active isolation devices and piezoelectric stack actuators. There are growing efforts between researchers to enhance structural performance through the application of smart systems and materials. However, further research is needed to determine the impact of these systems and materials on sustainability issues, particularly the long-term environmental impact of smart materials on human health and nature over their life cycle.

## Figures and Tables

**Table 1:** Comparison of physical and mechanical characteristics of Ni-Ti and Fe-based SMAs, and stainless steel [47].

Property	Unit ${}^{a}$	Ni-Ti ${}^{b}$	Fe-SMA	Steel
Density	kg/m${}^{3}$	6450–6500	7200–7500	7850
Corrosion resistance	-	Excellent	Good	Fair
Melting point	${}^{\circ }$C	1260–1310	1320–1350	1510
Poisson’s ratio	-	0.33	0.359	0.265
Young’s modulus	GPa	28–83	160–200	190–193
Specific heat capacity	J/kg ${}^{\circ }$C	450–620	540	420–510
Thermal conductivity	w/m ${}^{\circ }$C	8.6–18	8.4	8.9–16.2
Ultimate tensile strength	MPa	895–1900	680–1200	505
Yield Stress	MPa	70–690	475	215
Recoverable elongation	%	5–10	2.5–13	0.8
Elongation failure	%	5–50	12.4–20	20

${}^{a}$ The values for the given characteristics are based on the findings of many research, as indicated in the sources, to provide a wide variety of information; ${}^{b}$ the range of the provided values for Ni-Ti is determined by the crystal phase (martensite or austenite) and other factors (hardened or fully annealed).

**Table 2:** Comparison of several properties between main Cu-based and Ni-Ti SMAs.

Property	Unit	Ni-Ti	Cu-Based SMAs	
			Cu–Zn–Al	Cu–Al–Ni
Specific heat capacity	J/kg ${}^{\circ }$C	450–620	390–400	373–574
Thermal conductivity	W/m K	8.6–18	84–120	30–75
Density	kg/m${}^{3}$	6450–6500	7450–8000	7100–7200
Max. recovery stress	MPa	500–900	400–700	300–600
Fatigue strength	MPa	350	270	350
Normal number Of thermal cycles	-	105	104	5 × 10^3^
Young’s modulus	GPa	28–83	70–100	80–100
Transformation temperature range	${}^{\circ }$C	−200–200	−200–150	−200–200
Hysteresis	${}^{\circ }$C	2–50	5–20	20–40
Damping capacity	SDC%	15–20	30–85	10–20
Cost ratio	-	10–100	1–10	1.5–20
Workability	-	Fair	Good	Poor

**Table 3:** Comparison of properties between SMAs and SMPs [58].

Property	Unit	SMAs	SMPs
Density	kg/m${}^{3}$	6000–8000	900–1250
Transition breath	${}^{\circ }$C	5–30	10–50
Strain	%	Up to 40%	400–800
Young’s modulus	GPa	83 (Ni-Ti)	10–10,000
Deformation stress	MPa	50–200	1–3
Recovery stress	MPa	150–300	1–3
Recovery speed	S	Less than 1 s	Between 1 s to few minutes
Thermal conductivity	W/m K	18 (Ni–Ti, Austenite)	0.15–0.30 W/m K
Phase transformation	-	Martensitic transformation	Glass transition
Bio-compatibility	-	Medium	High
Corrosion resistance	-	Excellent	Excellent
High temperature’s condition	-	Hard	Soft
Low temperature’s condition	-	Soft	Hard
Cost	$/kg	550	22
Shape training	-	Difficult	Easy and fast
Fabrication/processing condition	-	1000 ${}^{\circ }$C, high pressure	200 ${}^{\circ }$C, Low pressure

**Table 4:** A comparison between the steel-based and SMA-based bracing systems [69].

Properties	Type of Bracing Frame	
	Steel-Based	SMA-Based
In-plane frequency (Hz)	35	35
Out-of-plane frequency (Hz)	39	46.8
Damping ratio (%)	1.3	1
Theoretical stiffness (kN/mm)	9.5	17.6
Experimental stiffness (kN/mm)	2.05	15.4

**Table 5:** The reduction percentage of the out-of-plane deformation in the SSW retrofitted with SMA [71].

Earthquakes	Reduction Due to SMA-Retrofitting		
	Max. Out-of-Plane Dis. (%)	Total Out-of-Plane Dis. In Shear Wall (%)	Reduction of Max. Stress (%)
Loma Prieta	12.73	23.41	−1.89
Landers	1	12.94	4.3
Gazli	44.43	15.26	1.16
Artificial	16.96	31.64	5.39

## Data Availability

The data presented in this article are available on request of the corresponding author.

## References

[1] Bazazzadeh H., Pilechiha P., Nadolny A., Mahdavinejad M., Hashemi safaei S.S. (2021). The Impact Assessment of Climate Change on Building Energy Consumption in Poland. Energies.

[2] Bazazzadeh H., Nadolny A., Safaei S.S.H. (2021). Climate Change and Building Energy Consumption: A Review of the Impact of Weather Parameters Influenced by Climate Change on Household Heating and Cooling Demands of Buildings. Eur. J. Sustain. Dev..

[3] Construction G. (2015). A Global Forecast for the Construction Industry to 2030.

[4] Lu W., Yuan H. (2011). A framework for understanding waste management studies in construction. Waste Manag..

[5] Burgan B.A., Sansom M.R. (2006). Sustainable steel construction. J. Construct. Steel Res..

[6] United Nations Office for Disaster Risk Reduction (UNISDR), Centre for Research on the Epidemiology of Disasters (CRED) (2018). Economic Losses, Poverty and Disasters 1998–2017.

[7] Goltermann P., Jensen F., Andersen M.E. Smart structures: Possibilities, experiences and benefits from permanent monitoring. Proceedings of the IABMAS.

[8] IŞik E. (2016). Consistency of the rapid assessment method for reinforced concrete buildings. Earthq. Struct..

[9] Javanmardi A., Ghaedi K., Huang F., Hanif M.U., Tabrizikahou A. (2021). Application of Structural Control Systems for the Cables of Cable-Stayed Bridges: State-of-the-Art and State-of-the-Practice. Arch. Comput. Methods Eng..

[10] Harirchian E., Kumari V., Jadhav K., Raj Das R., Rasulzade S., Lahmer T. (2020). A Machine Learning Framework for Assessing Seismic Hazard Safety of Reinforced Concrete Buildings. Appl. Sci..

[11] Daniell J.E., Shroder J.F., Wyss M. (2014). The Socioeconomic Impact of Earthquake Disasters. Earthquake Hazard, Risk and Disasters.

[12] Daniell J.E., Khazai B., Wenzel F., Vervaeck A. (2011). The CATDAT damaging earthquakes database. Nat. Hazards Earth Syst. Sci..

[13] Javanmardi A., Abadi R., Marsono A.K., Md Tap M., Ibrahim Z., Ahmad A. (2015). Correlation of Stiffness and Natural Frequency of Precast Frame System. Appl. Mech. Mater..

[14] Hou S., Li H., Rezgui Y. (2015). Ontology-based approach for structural design considering low embodied energy and carbon. Energy Build..

[15] Tabrizikahou A., Nowotarski P. (2021). Mitigating the Energy Consumption and the Carbon Emission in the Building Structures by Optimization of the Construction Processes. Energies.

[16] Jaganathan S., Nesan L.J., Ibrahim R., Mohammad A.H. (2013). Integrated design approach for improving architectural forms in industrialized building systems. Front. Archit. Res..

[17] Whang S.W., Kim S. (2015). Balanced sustainable implementation in the construction industry: The perspective of Korean contractors. Energy Build..

[18] Turskis Z., Urbonas K., Daniūnas A. (2019). A Hybrid Fuzzy Group Multi-Criteria Assessment of Structural Solutions of the Symmetric Frame Alternatives. Symmetry.

[19] Chopra I., Sirohi J. (2013). Smart Structures Theory.

[20] Sadasivuni K.K., Cabibihan J.J., Ponnamma D., AlMaadeed M.A., Kim J. (2016). Biopolymer Composites in Electronics.

[21] Gupta V., Sharma M., Thakur N. (2011). Mathematical modeling of actively controlled piezo smart structures: A review. Smart Struct. Syst..

[22] Alam M.S., Youssef M.A., Nehdi M. (2007). Utilizing shape memory alloys to enhance the performance and safety of civil infrastructure: A review. Can. J. Civ. Eng..

[23] Chang W.S., Araki Y. (2016). Use of shape-memory alloys in construction: A critical review. Proc. Inst. Civ. Eng. Civ. Eng..

[24] Ozbulut O.E., Hurlebaus S., Desroches R. (2011). Seismic Response Control Using Shape Memory Alloys: A Review. J. Intell. Mater. Syst. Struct..

[25] Zareie S., Issa A.S., Seethaler R.J., Zabihollah A. (2020). Recent advances in the applications of shape memory alloys in civil infrastructures: A review. Structures.

[26] Desroches R., Smith B. (2004). Shape memory alloys in seismic resistant design and retrofit: A critical review of their potential and limitations. J. Earthq. Eng..

[27] Naresh C., Bose P.S.C., Rao C.S.P. (2016). Shape memory alloys: A state of art review. IOP Conf. Ser. Mater. Sci. Eng..

[28] Song G., Ma N., Li H.N. (2006). Applications of shape memory alloys in civil structures. Eng. Struct..

[29] Wilson J.C., Wesolowsky M.J. (2005). Shape Memory Alloys for Seismic Response Modification: A State-of-the-Art Review. Earthq. Spectra.

[30] Dolce M., Cardone D. (2006). Theoretical and Experimental Studies for the Application of Shape Memory Alloys in Civil Engineering. J. Eng. Mater. Technol..

[31] Van Humbeeck J. (2001). Shape Memory Alloys: A Material and a Technology. Adv. Eng. Mater..

[32] Janke L., Czaderski C., Motavalli M., Ruth J. (2005). Applications of shape memory alloys in civil engineering structures—Overview, limits and new ideas. Mater. Struct..

[33] Cladera A., Weber B., Leinenbach C., Czaderski C., Shahverdi M., Motavalli M. (2014). Iron-based shape memory alloys for civil engineering structures: An overview. Construct. Build. Mater..

[34] Mohd Jani J., Leary M., Subic A., Gibson M.A. (2014). A review of shape memory alloy research, applications and opportunities. Mater. Des. (1980–2015).

[35] World Commission on Environment and Development (WCED) (1987). Report of the World Commission on Environment and Development: Our Common Future (The Brundtland Report). Med. Confl. Surv..

[36] Elkington J. (1998). Partnerships fromcannibals with forks: The triple bottom line of 21st-century business. Environ. Qual. Manag..

[37] Willard B. (2012). The New Sustainability Advantage: Seven Business Case Benefits of a Triple Bottom Line.

[38] Gencturk B., Hossain K., Lahourpour S. (2016). Life cycle sustainability assessment of RC buildings in seismic regions. Eng. Struct..

[39] Padgett J.E., Tapia C. (2013). Sustainability of Natural Hazard Risk Mitigation: Life Cycle Analysis of Environmental Indicators for Bridge Infrastructure. J. Infrastruct. Syst..

[40] Akiyama H., Teshigawara M., Fukuyama H. A framework of structural performance evaluation system for buildings in Japan. Proceedings of the 12th World Conference on Earthquake Engineering.

[41] Bogue R. (2009). Shape-memory materials: A review of technology and applications. Assem. Autom..

[42] Auricchio F., Taylor R.L., Lubliner J. (1997). Shape-memory alloys: Macromodelling and numerical simulations of the superelastic behavior. Comput. Methods Appl. Mech. Eng..

[43] Müller I., Xu H. (1991). On the pseudo-elastic hysteresis. Acta Metall. Mater..

[44] Raniecki B., Lexcellent C.T.K. (1992). Thermodynamic models of pseudoelastic behaviour of shape memory alloys. Arch. Mech..

[45] Kuczma M., Mielke A., Stein E. (1999). Modelling of hysteresis in two phase systems. Arch. Mech..

[46] Kuczma M., Mielke A. (2000). Influence of hardening and inhomogeneity on internal loops in pseudoelasticity. ZAMM.

[47] Tabrizikahou A., Hadzima-Nyarko M., Kuczma M., Lozančić S. (2021). Application of Shape Memory Alloys in Retrofitting of Masonry and Heritage Structures Based on Their Vulnerability Revealed in the Bam 2003 Earthquake. Materials.

[48] Ma J., Hornbuckle B., Karaman I., Thompson G., Luo Z., Chumlyakov Y. (2013). The effect of nanoprecipitates on the superelastic properties of FeNiCoAlTa shape memory alloy single crystals. Acta Mater..

[49] Omori T., Ando K., Okano M., Xu X., Tanaka Y., Ohnuma I., Kainuma R., Ishida K. (2011). Superelastic Effect in Polycrystalline Ferrous Alloys. Science.

[50] Cladera A., Montoya-Coronado L.A., Ruiz-Pinilla J.G., Ribas C. (2020). Shear strengthening of slender reinforced concrete T-shaped beams using iron-based shape memory alloy strips. Eng. Struct..

[51] Lee W.J., Weber B., Feltrin G., Czaderski C., Motavalli M., Leinenbach C. (2013). Phase transformation behavior under uniaxial deformation of an Fe-Mn-Si-Cr-Ni-VC shape memory alloy. Mater. Sci. Eng. A.

[52] Dong Z., Klotz U.E., Leinenbach C., Bergamini A., Czaderski C., Motavalli M. (2009). A Novel Fe-Mn-Si Shape Memory Alloy With Improved Shape Recovery Properties by VC Precipitation. Adv. Eng. Mater..

[53] Leinenbach C., Kramer H., Bernhard C., Eifler D. (2012). Thermo-Mechanical Properties of an Fe-Mn-Si-Cr-Ni-VC Shape Memory Alloy with Low Transformation Temperature. Adv. Eng. Mater..

[54] Shahverdi M., Michels J., Czaderski C., Motavalli M. (2018). Iron-based shape memory alloy strips for strengthening RC members: Material behavior and characterization. Construct. Build. Mater..

[55] Dasgupta R. (2014). A look into Cu-based shape memory alloys: Present scenario and future prospects. J. Mater. Res..

[56] Sutou Y., Omori T., Kainuma R., Ishida K. (2008). Ductile Cu–Al–Mn based shape memory alloys: General properties and applications. Mater. Sci. Technol..

[57] Meng Q., Hu J. (2009). A review of shape memory polymer composites and blends. Compos. Part A Appl. Sci. Manuf..

[58] Hornbogen E. (2006). Comparison of shape memory metals and polymers. Adv. Eng. Mater..

[59] Wilkes K.E., Liaw P.K., Wilkes K.E. (2000). The fatigue behavior of shape-memory alloys. JOM.

[60] Dolatabadi N., Shahverdi M., Ghassemieh M., Motavalli M. (2020). RC Structures Strengthened by an Iron-Based Shape Memory Alloy Embedded in a Shotcrete Layer—Nonlinear Finite Element Modeling. Materials.

[61] Schranz B., Michels J., Czaderski C., Motavalli M., Vogel T., Shahverdi M. (2021). Strengthening and prestressing of bridge decks with ribbed iron-based shape memory alloy bars. Eng. Struct..

[62] Czaderski C., Shahverdi M., Michels J. (2021). Iron based shape memory alloys as shear reinforcement for bridge girders. Construct. Build. Mater..

[63] Song G., Mo Y.L., Otero K., Gu H. (2006). Health monitoring and rehabilitation of a concrete structure using intelligent materials. Smart Mater. Struct..

[64] Youssef M.A., Alam M.S., Nehdi M. (2008). Experimental Investigation on the Seismic Behavior of Beam-Column Joints Reinforced with Superelastic Shape Memory Alloys. J. Earthq. Eng..

[65] Alam M., Youssef M., Nehdi M. (2008). Analytical prediction of the seismic behaviour of superelastic shape memory alloy reinforced concrete elements. Eng. Struct..

[66] Hedayati Dezfuli F., Alam M.S. (2018). Smart Lead Rubber Bearings Equipped with Ferrous Shape Memory Alloy Wires for Seismically Isolating Highway Bridges. J. Earthq. Eng..

[67] Billah A.M., Alam M.S. (2018). Probabilistic seismic risk assessment of concrete bridge piers reinforced with different types of shape memory alloys. Eng. Struct..

[68] Andrawes B., Shin M., Wierschem N. (2010). Active Confinement of Reinforced Concrete Bridge Columns Using Shape Memory Alloys. J. Bridge Eng..

[69] Varughese K., El-Hacha R. (2021). Experimental free vibrations test of steel braced frames reinforced with NiTi shape memory alloy wires. Structures.

[70] Shi F., Zhou Y., Ozbulut O.E., Cao S. (2021). Development and experimental validation of anchorage systems for shape memory alloy cables. Eng. Struct..

[71] Kamgar R., Heidarzadeh H., Babadaei Samani M.R. (2021). Evaluation of buckling load and dynamic performance of steel shear wall retrofitted with strips made of shape memory alloy. Sci. Iran..

[72] Wang B., Zhu S., Qiu C.X., Jin H. (2019). High-performance self-centering steel columns with shape memory alloy bolts: Design procedure and experimental evaluation. Eng. Struct..

[73] Liu Y., Wang H., Qiu C., Zhao X. (2019). Seismic behavior of superelastic shape memory alloy spring in base isolation system of multi-story steel frame. Materials.

[74] Hadzima-Nyarko M., Mišetić V., Morić D. (2017). Seismic vulnerability assessment of an old historical masonry building in Osijek, Croatia, using Damage Index. J. Cult. Herit..

[75] Hadzima-Nyarko M., Pavić G., Lešić M. (2016). Seismic vulnerability of old confined masonry buildings in Osijek, Croatia. Earthq. Struct..

[76] Cardone D., Angiuli R., Gesualdi G. (2019). Application of Shape Memory Alloys in Historical Constructions. Int. J. Archit. Herit..

[77] Bazazzadeh H., Nadolny A., Attarian K., Safar ali najar B., sara Hashemi safaei S. (2020). Promoting Sustainable Development of Cultural Assets by Improving Users’ Perception through Space Configuration; Case Study: The Industrial Heritage Site. Sustainability.

[78] Mahdavinejad M., Didehban M., Bazazzadeh H. (2016). Contemporary architectural heritage and industrial identity in historic districts, case study: Dezful. J. Stud. Iran. Islamic City.

[79] Casciati S., Hamdaoui K. (2008). Experimental and numerical studies toward the implementation of shape memory alloy ties in masonry structures. Smart Struct. Syst..

[80] Habieb A.B., Valente M., Milani G. (2019). Hybrid seismic base isolation of a historical masonry church using unbonded fiber reinforced elastomeric isolators and shape memory alloy wires. Eng. Struct..

[81] Rezapour M., Ghassemieh M., Motavalli M., Shahverdi M. (2021). Numerical Modeling of Unreinforced Masonry Walls Strengthened with Fe-Based Shape Memory Alloy Strips. Materials.

[82] Castellano M.G., Indirli M., Martelli A. (2001). Progress of application, research and development, and design guidelines for shape memory alloy devices for cultural heritage structures in Italy. Smart Structures and Materials 2001: Smart Systems for Bridges, Structures, And Highways.

[83] Chen G., Chen C., Liu S.C. (2000). Behavior of piezoelectric friction dampers under dynamic loading. Smart Structures and Materials 2000: Smart Systems for Bridges, Structures, and Highways.

[84] Aizawa S., Kakizawa T., Higasino M. (1998). Case studies of smart materials for civil structures. Smart Mater. Struct..

[85] Kamada T., Fujita T., Hatayama T., Arikabe T., Murai N., Aizawa S., Tohyama K. (1997). Active vibration control of frame structures with smart structures using piezoelectric actuators (Vibration control by control of bending moments of columns). Smart Mater. Struct..

[86] Kamada T., Fujita T., Hatayama T., Arikabe T., Murai N., Aizawa S., Tohyama K. (1998). Active vibration control of flexural-shear type frame structures with smart structures using piezoelectric actuators. Smart Mater. Struct..

[87] Morita K., Fujita T., Ise S., ichi Kawaguchi K., Kamada T., Fujitani H., Liu S.C. (2001). Development and application of induced-strain actuators for building structures. Smart Structures and Materials 2001: Smart Systems for Bridges, Structures, and Highways.

[88] Challa V.R., Prasad M.G., Shi Y., Fisher F.T. (2008). A vibration energy harvesting device with bidirectional resonance frequency tunability. Smart Mater. Struct..

[89] Maamer B., Boughamoura A., Fath El-Bab A.M., Francis L.A., Tounsi F. (2019). A review on design improvements and techniques for mechanical energy harvesting using piezoelectric and electromagnetic schemes. Energy Convers. Manag..

[90] Morris D.J., Youngsman J.M., Anderson M.J., Bahr D.F. (2008). A resonant frequency tunable, extensional mode piezoelectric vibration harvesting mechanism. Smart Mater. Struct..

